# Task Offloading Algorithm for Multiple Unmanned Aerial Vehicles Based on Temporal Graph

**DOI:** 10.3390/s25216759

**Published:** 2025-11-05

**Authors:** Lingyu Zhao, Xiaorong Zhu, Jianhong Cai

**Affiliations:** College of Telecommunications and Information Engineering, Nanjing University of Posts and Telecommunications, Nanjing 210003, China; 2020010201@njupt.edu.cn (L.Z.);

**Keywords:** computing network, task offloading, temporal graph, two-stage matching algorithm, UAV

## Abstract

With the rapid expansion of data scale, compute-intensive tasks will become a core application of 6G networks. As Unmanned Aerial Vehicle (UAV) technology advances, UAVs can assist in task offloading for mobile edge computing by collaborating to overcome individual UAV limitations in battery life and computational capacity. Hence, in this paper, we propose a task offloading algorithm for multiple UAVs based on a temporal graph. We first formulate an optimization problem to minimize the total completion time of UAV swarm task offloading by classifying tasks and determining task priorities and subtask dependencies. To solve this problem, we introduce a temporal graph to simulate service nodes and task sequences in computing networks. It can reveal task execution priorities by calculating proximity indices, which indicate the ratio of physical distance to the sum of task weights, and determining timestamp offsets. In the following, to reduce unnecessary waiting and computation resource allocation risks, we transform the optimization problem into a directed acyclic graph connectivity problem, which identifies the fastest temporal paths for each UAV, forming a dedicated service network. Finally, we propose a two-stage matching algorithm that achieves optimal matching based on service node locations, statuses, task types, and offloading demands. Simulation results demonstrate that the algorithm performs exceptionally well, reducing task completion times and significantly outperforming other algorithms in terms of task utility.

## 1. Introduction

With the diversification of network services, computationally intensive applications are gradually becoming the mainstream of 6G network applications [1]. These applications are characterized by high-quality service requirements, substantial computational resource demands, and the need for high-performance hardware support [2]. However, user devices have limited capabilities in terms of computility (Computility is the ability to solve practical problems with computing resources. For example, in cloud computing and distributed systems, it not only involves dynamically adjusting cloud server capacity to balance performance and cost but also using edge computing to cut latency and boost real-time utility.) and energy, thus necessitating additional computational resources provided by edge computing.

Mobile Edge Computing (MEC) is an architecture that places computing power, storage, and data processing at the edge of mobile networks (near users and devices) [3,4]. It has become one of the key technologies for 6G networks. By shortening data transmission distances and optimizing local processing, it greatly reduces latency, improves efficiency, and supports applications with high real-time demands. The MEC architecture can alleviate the load on cloud servers, thereby reducing network congestion and latency and enhancing user experience. As data processing and storage are performed at the edge devices, this not only increases data security but also protects user privacy [5]. However, compared to centralized cloud centers, edge devices are more widely distributed and challenging to maintain and manage. Additionally, due to limited computing resources, edge devices often struggle to meet the diverse computational demands of various tasks simultaneously. Therefore, we need to develop effective task offloading strategies to address the growing number of compute-intensive tasks.

To overcome these limitations, the integration of unmanned aerial vehicles (UAVs) offers a new solution for edge computing [6]. UAVs are autonomous flying vehicles characterized by their high mobility, rapid deployment capabilities, and relatively low cost [7]. Currently, they have found extensive applications in fields such as aerial photography, agriculture, package delivery, disaster relief, mapping, news reporting, and power line inspections. UAVs can be deployed as aerial edge servers or relays, thereby enhancing edge computing services. In contrast to conventional MEC networks, aerial UAV networks face unique challenges such as high mobility, task heterogeneity, and real-time offloading demands. These challenges require the development of specialized offloading algorithms, such as the temporal graph-based approach proposed in this work, to optimize task completion times in dynamic environments. Computing tasks are generated at target points, which are typically end devices such as sensors and cameras. These tasks, based on mission objectives such as surveillance, environmental monitoring, or disaster recovery, are then scheduled for computation at the edge nodes. These edge nodes include UAVs acting as relays or MEC servers. The edge computing architecture facilitates the offloading of computational tasks from the UAVs to these edge nodes, which reduces latency and improves processing efficiency. However, model constraints like service caching, resource capacity, task dependencies, and delay thresholds are used independently and lack joint optimization within a unified framework.

Graph theory, the fundamental theory of complex networks, can effectively address the above problems. By building a bidirectional preference model between subtask requirements (e.g., computing type, caching dependency, priority) and edge node attributes (e.g., service caching status, residual resources, topological location), matching algorithms can achieve fine-grained supply–demand matching. Also, to maximize collaborative task offloading effectiveness for multiple UAVs, it classifies task types and those supported by UAVs. To our knowledge, we are the first to use temporal graphs and two-stage matching algorithms to solve the problem of UAV task offloading. The contributions of this work are summarized as follows:

- We establish an optimization problem aimed at minimizing the total task completion time for UAV swarms. To model this problem, we introduce a temporal graph to simulate service nodes and task sequences in computing networks. When manually constructing timestamps on the edges of a temporal graph, we determine the offset based on the proximity index ranking of conflicts at each service node and set the basic quantity based on path hops. The temporal sequence represented by the timestamps reveals the execution order among tasks.
- We transform the optimization problem into a connectivity issue in graph theory from a graph-theoretical perspective to reduce unnecessary waiting and computation resource allocation risks. Then, using total traversal time as a metric, we find the fastest temporal graph path for each UAV within the temporal graph and assign the set of service nodes included in the path to form an exclusive service network for UAV.
- We propose a two-stage matching algorithm for the task offloading strategy problem. Given the UAV service network and tasks to offload, we establish the bilateral supply–demand relationship between tasks and the service network based on task classification and dependencies. We complete the overall matching considering the actual service node locations, service node status, task types, and task offloading demands.
- We evaluate the proposed task offloading algorithm based on the exchange matching algorithm through experimental simulation. The results show that this algorithm excels in optimizing the total task completion time for a swarm of UAVs, validating its effectiveness.

The remainder of this paper is organized as follows: Section 2 reviews the related work and highlights the novelties of this paper. Section 3 provides a detailed description of the system model and formulates the optimization problem; Section 4 introduces our task offloading algorithm based on the exchange matching method; Section 5 presents performance analysis and discussion; Section 6 summarizes our works; Section 7 highlights potential extensions and open challenges.

## 2. Related Work

Research on UAV-assisted edge computing has expanded rapidly in recent years, driven by the growing demand for resilient, low-latency computation in scenarios such as disaster recovery, emergency communications, and real-time data processing. Table 1 summarizes and compares representative studies with our proposed work. Broadly, prior research can be categorized into three dimensions: (i) UAV-based task offloading and edge integration; (ii) task heterogeneity and collaborative execution strategies; (iii) offloading optimization under task dependency constraints; (iv) graph-based modeling for task offloading.

### 2.1. UAV-Based Task Offloading and Edge Integration

In [8], the authors propose using UAVs and high-altitude platforms to establish temporary post-disaster communication networks. Their approach divides the process into task collection and task offloading, enabling coverage restoration in disrupted areas. Existing research highlights the effectiveness of UAVs in re-establishing communication infrastructure and providing task offloading capabilities; however, the synergy between UAV operations and MEC architectures remains an essential enabler for scalable and robust solutions [9].

Further, multi-objective optimization has been introduced to better leverage UAV capabilities. Sun et al. [10] converts a multi-objective problem into a single-objective formulation, demonstrating improved performance in heterogeneous UAV environments. In [11], tasks are modeled across multiple dimensions, and UAVs are integrated into MEC systems to improve response speed through flexible task offloading strategies, ultimately mitigating network congestion and optimizing resource use. Additionally, Huang et al. [12] addresses multi-type UAV collaborative task assignment under resource constraints, significantly enhancing resource utilization and task completion quality. Li et al. [13] proposes a learning-driven stochastic game approach with adaptive clustering and scheduling to enhance energy efficiency and reduce delay in UAV-assisted MEC systems. Differently, our method leverages temporal graphs for better task scheduling and offloading optimization and incorporates a two-stage matching algorithm, which is designed to handle time-dependent task assignments in dynamic UAV swarm environments, making it more adaptable to real-world scenarios.

### 2.2. Task Heterogeneity and Collaborative Execution

The challenge of task heterogeneity has been explored by works such as [14], which investigates multi-UAV task collaboration in environments with diverse task types and requirements. The authors show that certain tasks may require multiple UAVs to cooperate, forming ad hoc teams to complete complex objectives. Similarly, Qi et al. [15] studies alliance formation in heterogeneous UAV networks, where tasks consist of multiple subtasks categorized by type, and alliances jointly execute these subtasks. In the context of UAV swarms, Li et al. [16] considers the dynamic allocation of tasks and resources using a task sequence mechanism, differentiating between electronic jamming tasks and attack tasks to improve mission outcomes. Although these studies account for heterogeneous tasks, they typically do not model the intrinsic order of task execution, which is critical in scenarios like disaster response, where steps such as human detection must precede victim localization. Addressing this temporal ordering is crucial for improving operational efficiency and ensuring mission-critical tasks are prioritized correctly.

### 2.3. Task Dependency-Aware Offloading

Beyond macro-level task sequencing, subtasks often exhibit fine-grained dependencies that must be respected during offloading and execution [17]. This creates a dual-layer optimization challenge involving both task scheduling and resource allocation. In [18], the authors examine the offloading of dependent tasks with service caching, formulating an optimization model with delay minimization as the objective and proving its computational complexity. Their proposed convex-approximation algorithm provides near-optimal performance in managing task execution at specific edge nodes where required services are cached. Similarly, Liu et al. [19] investigates multi-application scheduling and shows that the problem is NP-hard. By prioritizing subtasks according to their dependency chains, the authors reduce overall completion time by assigning critical subtasks to edge nodes capable of minimizing latency. Complementing these dependency-aware strategies, Yang et al. [20] introduces a two-timescale online optimization method for dynamically managing human digital twins at the edge, improving task accuracy while balancing latency and energy use. In contrast, our method leverages a spatio-temporal graph and a two-stage matching algorithm, which provides distinct advantages by modeling temporal dependencies and task sequencing. This makes our method more adaptable to real-time, dynamic environments and UAV mobility, enabling more flexible and scalable solutions in networks.

### 2.4. Graph-Based Modeling for Task Offloading

To better represent task offloading, Holme et al. [21] introduces a time dimension, marking each edge’s timestamp in the graph to show interaction moments or periods between vertices, forming a complete temporal graph. Li et al. [22] introduces GASTO, a fast and adaptive graph learning framework that dynamically constructs task–network representations to improve offloading efficiency. Cai et al. [23] introduces a mobility-aware offloading scheme using temporal graphs and graph matching, effectively handling dynamic user mobility and task dependencies to enhance task utility and success rates in 6G edge computing environments. Yan et al. [24] presents a multiendpoint DAG-based optimization framework that jointly addresses partitioning, offloading, and scheduling for distributed DNN inference, significantly reducing latency and improving scalability across heterogeneous edge networks. Differently, our method introduces a unified graph-theoretic framework that jointly incorporates temporal dynamics and task dependencies, enabling simultaneous improvements in offloading efficiency, resource utilization, and adaptability across heterogeneous edge environments.

**Table 1:** Comparison of related work with the proposed work.

Reference	Multiple UAVs-MEC	Task Completion Time	Task Classification	Task Dependency	Graph Theory
Wang et al. [7]	**✗**	**✔**	**✗**	**✗**	**✗**
Kang et al. [8]	**✗**	**✔**	**✗**	**✗**	**✗**
Sun et al. [10]	**✔**	**✔**	**✗**	**✗**	**✗**
Akter et al. [11]	**✔**	**✔**	**✗**	**✗**	**✗**
Huang et al. [12]	**✗**	**✗**	**✔**	**✗**	**✗**
Li et al. [13]	**✔**	**✔**	**✗**	**✗**	**✗**
Qi et al. [15]	**✗**	**✗**	**✗**	**✔**	**✗**
Fu et al. [16]	**✗**	**✔**	**✗**	**✗**	**✗**
Yang et al. [20]	**✗**	**✔**	**✗**	**✗**	**✔**
Cai et al. [23]	**✗**	**✔**	**✗**	**✔**	**✔**
Our paper	**✔**	**✔**	**✔**	**✔**	**✔**

## 3. System Model

### 3.1. System Description

**Figure 1:**
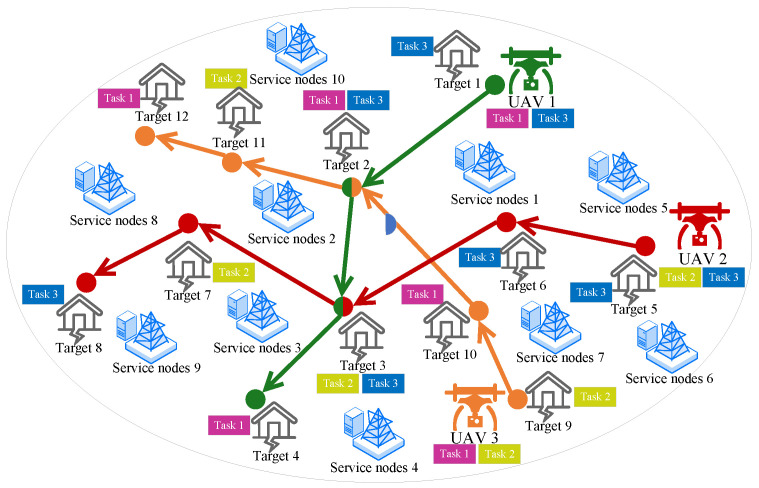
Scenario diagram of multi-UAV executing tasks.

As depicted in Figure 1, the system consists of target locations under surveillance, and multiple UAVs collaborate to perform reconnaissance tasks. To accomplish the reconnaissance mission in the target area, we assign different tasks to various UAVs. Prior to departure, each UAV has determined the sequence of target locations to be surveyed [25] and the tasks to be executed at each location. Subsequently, the UAVs must offload the collected information to edge servers within the area to obtain computational results. All important notations and their corresponding definitions are presented in Table 2.

We denote the set of tasks in the system as $X=\{x_{1},x_{2},\ldots ,x_{N}\}$, where tasks of type $x_{n}$ are executed before tasks of type $x_{n-1}$. The set of task types supported by UAV *m* is $X_{m}\subseteq X$, where m∈M={1,2,…,M}. At the target location k∈K={1,2,…,K} within the surveillance area, the set of task types that the UAV must perform is $X_{k}\subseteq X$. The pre-assigned task location set for UAV *m* is denoted as $K^{m}\subseteq K$, and its predetermined route for task processing is $Q_{m}$. The set of routes for all UAVs is denoted as Q, with $Q_{m}\in Q$. Since each UAV has been pre-assigned a set route and task set, we consider the available MEC servers within the area as potential service nodes s∈S={1,2,…,S}. The set of service nodes available to UAV *m* throughout its movement is $S^{m}\subseteq S$.

Assuming the system operates in time slots, each time slot t∈T={1,2,…,T} has a length of $\tau_{0}$. If a task generated at a location cannot be completed within a single time slot, it is divided into multiple subtasks to be accomplished within a single time slot. If a task at a location consists of interdependent subtasks, the offloading order is first determined based on the dependencies among the subtasks, and then these subtasks are offloaded in this order across different time slots. We assume that there is only one task of the same type at each location.

### 3.2. Task Offloading Model

Assuming UAV *m* collects a task at location $k^{m}$, denoted as $D_{m,k^{m}}=\left\{\mu_{m,k^{m}},\lambda_{m,k^{m}}\right\}$ [26], where $\mu_{m,k^{m}}$[cycles/bit] represents the computational resources required for the task, and $\lambda_{m,k^{m}}$[bit] represents the input data volume of the task. We denote the set of tasks collected by UAV *m* during the time period [1,T] as $D_{m}=\sum_{k=1}^{K}D_{m,k^{m}}$, and the set of tasks collected by all UAVs in the system as $D={⋃}_{m=1}^{M}D_{m}$. We model offloading tasks as a directed acyclic graph (DAG) $G_{D}=\left(V_{D},E_{D}\right)$. $V_{D}$ represents the set of subtasks, and edge set $E_{D}$ ($E_{D}\subseteq V_{D}\times V_{D}\times L_{D}$) represents dependencies between them. Edge labels $L_{D}$ denote the data volume transferred between two subtasks. Assuming the outcome of subtask $v_{i}\in V_{D}$ serves as the input for subtask $v_{j}\in V_{D}$, this indicates an interdependency $\left(v_{i},v_{j}\right)\in E_{D}$ between $v_{i}$ and $v_{j}$. Here, $v_{i}$ precedes $v_{j}$, making it the predecessor, while $v_{j}$ follows $v_{i}$, establishing it as the successor. Note that $v_{i}\left(v_{j}\right)$ only represents subtasks in $G_{D}$.

Considering that UAV *m* needs to offload tasks collected from different locations to suitable edge servers within the area, we represent the task offloading plan for UAV *m* during the time period [1,T] as a binary matrix $O_{m}$ of dimension $K^{m}\times S^{m}$. The binary matrix element $O_{m}\left(k^{m},s^{m}\right)$ indicates whether UAV *m* offloads the task $D_{m,k}$ generated at location $k^{m}$ to service node $s^{m}\in S^{m}$, with a value of 1 indicating offloading, and 0 otherwise. When the task offloading plan for UAV *m* is represented as a binary matrix $O_{m}$, the task offloading plan for all UAVs in the system can be represented as a binary matrix O of dimension |D|×S.

Note that due to the memory constraints of UAVs, the number of pending offloading tasks stored in a UAV should not exceed this limit. We have the following storage capacity constraints:

(1) $$\sum_{s=1}^{S^{m}}O_{m}\left(k^{m},s^{m}\right)\lambda_{m,k^{m}}\leq \Phi^{\max},$$

where $\Phi^{\max}$ represents the maximum cache capacity of the UAV.

When UAV *m* processes the task $D_{m,k^{m}}$ generated at location $k^{m}$, the total delay in completing the task includes flight delay $t_{m,s^{m}}^{\mathrm{fly}}$, collection delay $t_{m,s^{m}}^{\mathrm{col}}$, upload delay $t_{m,s^{m}}^{\mathrm{upload}}$, and computation delay $t_{m,s^{m}}^{\mathrm{comp}}$. The flight delay is the duration it takes for UAV *m* to fly from its current position to the next location point, which depends on the speed of UAV *m* and the distance between the two points. The collection delay refers to the time it takes for UAV *m* to collect a specified task after reaching location point $k^{m}$. The upload delay is the data transmission time required by UAV *m* to offload the task onto a service node. The computation delay is the time taken by the service node to process the task once it has been offloaded. Considering the small amount of output data after task processing, we neglect the backhaul delay of transmitting the computation results. Furthermore, for task $D_{m,k^{m}}$, the flight delay is primarily influenced by the predetermined task assignment scheme, which dictates the flight path of the UAV and directly determines the flight delay given a fixed speed. The collection delay depends on the processing capability of the equipment aboard the UAV. Pre-assigned routes and task sets simplify the modeling process and allow us to focus on the core aspects of offloading. Relaxing these assumptions increases model complexity and introduces higher computational costs. Considering these factors and focusing on our research objective of task offloading strategies, we will focus specifically on the transmission delay and computation delay of tasks.

Assuming that the spectrum resource allocated by service node $s^{m}$ to UAV *m* is $W_{m,s^{m}}$, the transmission power of UAV *m* is $p_{m}$, and the noise power spectrum of the channel is $\sigma_{0}$ (Since different service nodes use different communication frequencies, there is no mutual interference between channels.), the communication rate between UAV *m* and its service node $s^{m}$ is

(2) $$R_{m,s^{m}}=W_{m,s^{m}}\log_{2}\left(1+\frac{p_{m}\cdot g_{m,s^{m}}}{\sigma_{0}\cdot W_{m,s^{m}}}\right).$$

where $g_{m,s^{m}}$ is the channel gain between UAV *m* and service node $s^{m}$, it can be expressed as $g_{m,s^{m}}=\zeta_{\mathrm{ave}}\left(\mathrm{dB}\right)\cdot d_{m,s^{m}}^{-\alpha }$. $d_{m,s^{m}}^{-\alpha }$ is the distance between UAV *m* and service node $s^{m}$. α is the path loss exponent. $\zeta_{\mathrm{ave}}\left(\mathrm{dB}\right)$ is the average path loss between UAV *m* and service node $s^{m}$, which can be obtained by:

(3) $$\begin{array}{c} \zeta_{\mathrm{ave}}\left(\mathrm{dB}\right)=\rho_{\mathrm{LoS}}\zeta_{\mathrm{LoS}}+\rho_{\mathrm{NLoS}}\zeta_{\mathrm{NLoS}} \end{array}$$

where $\zeta_{\mathrm{LoS}}$ and $\zeta_{\mathrm{NLoS}}$ respectively represent the path loss of LoS and NLoS links [27]. $\rho_{\mathrm{LoS}}$ is the connection probability of the LoS link between the UAV and the service node, which can be expressed as

(4) $$\rho_{\mathrm{LoS}}=\frac{1}{1+a\exp(-b\left(\left(\frac{180}{\pi }\right)\arctan\left(\frac{h_{m}}{d_{m,s^{m}}}\right)-a\right))},$$

where *a* and *b* are two parameters related to the environment, $h_{m}$ is the flight height of the UAV. Similarly, the connection probability of the NLoS link is $\rho_{\mathrm{NLoS}}=1-\rho_{\mathrm{LoS}}$.

When calculating the task upload delay, we consider two scenarios: one where the task can be offloaded completely and another where it needs to be split into subtasks. In the first scenario, when the task can be offloaded completely, the input data volume for task $D_{m,k^{m}}$ is $\lambda_{m,k^{m}}$. In the second scenario, when the task needs to be split into subtasks, the data transfer volume between subtask $v_{j}$ and its preceding subtask $v_{i}$ is $\lambda_{i,j}\in L_{D}$. Assuming the set of preceding subtasks for subtask $v_{j}$ is denoted as $\mathrm{pred}(v_{j})$, the upload delay for subtask $v_{j}$ is the maximum data transfer delay between subtask $v_{j}$ and its preceding subtasks. In both scenarios, the communication rate between UAV *m* and service node $s^{m}$ is $R_{m,s^{m}}$, and the upload delay can be represented as

(5) $$t_{m,s^{m}}^{\mathrm{upload}}=\left\{\begin{array}{cc} \; & \frac{\lambda_{m,k^{m}}}{R_{m,s^{m}}},\;\mathrm{task}\;\mathrm{completely}\;\mathrm{offloaded},\\ \; & \max_{v_{i}\in \mathrm{pred}\left(v_{j}\right)}\frac{\lambda_{i,j}}{R_{m,s^{m}}},\;\mathrm{otherwise}. \end{array}\right.$$

Assuming that the computational resource allocated by service node $s^{m}$ to UAV *m* is $C_{m,s^{m}}$, then when the task $D_{m,k^{m}}$ requires computational resources $\mu_{m,k^{m}}$, the computation delay for this task is

(6) $$t_{m,s^{m}}^{\mathrm{comp}}=\frac{\mu_{m,k^{m}}}{C_{m,s^{m}}}.$$

When the upload delay for the task is $t_{m,s^{m}}^{\mathrm{upload}}$ and the computation delay is $t_{m,s^{m}}^{\mathrm{comp}}$, the completion delay for task $D_{m,k^{m}}$ is

(7) $$t_{m,k^{m}}=t_{m,s^{m}}^{\mathrm{upload}}+t_{m,s^{m}}^{\mathrm{comp}}.$$

### 3.3. Optimization Problem

Our objective is to enable collaborative multi-UAV to rapidly complete full-coverage reconnaissance missions in target areas through coordinated task execution. When the sequence of locations to be surveyed by UAV *m* is $K^{m}=1^{m},2^{m},\ldots ,K^{m}$, the completion delay for the task at the last location is $t_{m,K^{m}}$. When multiple UAVs collaborate to survey the target area, the total delay for this reconnaissance task should be the maximum completion delay of the last task for all UAVs, i.e., $\max(t_{1,k^{1}},t_{2,k^{2}},\ldots ,t_{M,k^{M}})$. To minimize the task completion delay, we formulate the following optimization problem:

(8) $$\begin{array}{cc} \; & \min_{O}\max\left(t_{1,k^{1}},t_{2,k^{2}},\ldots ,t_{M,k^{M}}\right),\\ s.t.\;\; & C1:O_{m}\left(k^{m},s^{m}\right)=\left\{0,1\right\},\\ \; & C2:\sum_{s=1}^{S^{m}}O_{m}\left(k^{m},s^{m}\right)=1,\\ \; & C3:\sum_{s=1}^{S^{m}}O_{m}\left(k^{m},s^{m}\right)\lambda_{m,k^{m}}\leq \Phi^{\max},\\ \; & \forall k^{m}\in K^{m},\forall s^{m}\in S^{m},\forall m\in M. \end{array}$$

where C1 indicates that the task offloading strategy is a binary variable, C2 indicates that any task collected by a UAV at any location can only be offloaded to one service node. C3 is the constraint on the UAV’s task caching. The objective function in Equation (8) presents mathematical challenges due to the non-linearity induced by the UAV flight paths and task dependencies. These complexities are handled by formulating the problem as a DAG, where tasks are modeled as nodes and their dependencies as edges. Task completion time is then minimized subject to various constraints, including UAV energy consumption and network bandwidth.

## 4. Task Offloading Algorithm Based on Temporal Graph

### 4.1. Methodology

Consider the following example: In a scenario where multiple UAVs are competing for a service node, the proximity index is calculated by summing the task weights of all tasks offloaded at that node and comparing them against the physical distances. The UAV with the smallest proximity index is granted the earliest timestamp, ensuring that tasks are offloaded in an efficient order.

#### 4.1.1. Establish the Temporal Graph

To construct a directed temporal graph $G_{S}=\left(V_{S},E_{S}\right)$, it is necessary to determine its set of nodes, set of edges, and the timestamps on each edge. The node set $V_{S}$ represents all available service nodes within the area under surveillance. UAVs can offload tasks to any available service node within the area. Hence, the node set of the temporal graph is denoted as $V_{S}=S=\left\{1,2,\ldots ,S\right\}$. It should be noted that UAVs only need to offload tasks and obtain information at pre-assigned locations, without the requirement to offload all tasks sequentially. In other words, if there are no suitable or nearby service nodes when a UAV is ready to offload a task at a particular location point, it can temporarily store the task without immediately offloading and processing it. Subsequently, the UAV can choose to continue processing the next task at another location point or seek out an appropriate service node during its journey to the next location point for task offloading. Thus, UAVs can process and offload tasks separately before performing a unified offload. It can also collect tasks from multiple points without exceeding its cache limit. This validates the rationality of using all available service nodes as a set of nodes.

Before establishing connections between nodes, we first need to clarify the role of the edge set $E_{S}$ in UAV task offloading. We abstract each UAV’s task offloading strategy into a directed path. The nodes on this path represent the set of service nodes where tasks are to be offloaded, and the order of the service nodes determines the sequence of task offloading. Additionally, the direction of the edges must align with the predefined flight direction of the UAVs. Therefore, when establishing connections, we can traverse all UAVs and determine the available service nodes near each location point based on their predetermined routes. We then select appropriate nodes to establish connection relationships. For instance, for UAV *m*, assuming its route passes through location points $2^{m}$ and $3^{m}$, if $\left(2^{m},3^{m}\right)\in Q_{m}$, the sets of nearby service nodes are $S_{2^{m}}$ and $S_{3^{m}}$. In this case, we choose any service node $S_{k-1}$ from $S_{2^{m}}$ and connect it to any service node $S_{k}$ from $S_{3^{m}}$, forming an edge $e_{k-1,k}\in E_{S}$. The weight of edge $e_{k-1,k}$ is the physical distance between the two service nodes, $w_{k-1,k}=\mathrm{dist}\left(S_{k-1},S_{k}\right)$, and its label is defined by the UAV’s identifier *m*, i.e., ${\mathrm{label}}_{k-1,k}=m$ (This variable will be used to determine the timestamps of the edges later).

In the temporal graph, timestamps can be used to represent the moments or periods when two entities interact, as well as to represent temporal graph paths (i.e., paths where timestamps do not decrease). Since we cannot obtain the exact time points at which a UAV is at each service node, the start and end times of the timestamps are unknown. This means that we cannot define the timestamps to represent specific moments or periods when two entities interact. Therefore, we can only use timestamps to form temporal graph paths within the temporal graph and represent the order of interactions through their chronological sequence.

There are two types of precedence constraints: the first case is the predetermined flight route for each UAV; the directed nature of edges in the temporal graph ensures the order of the UAVs’ flight routes. The second case involves using edge timestamps to represent the execution order of multiple tasks of different types at each location point. Since a single location point can have multiple task types, these tasks are handled by different UAVs and may be offloaded to the same service node near that location point. To avoid access conflicts (reduce competition for the same service node among multiple UAVs.), we plan to design timestamps reasonably so that these UAVs access the service nodes in both time and space, achieving a staggered usage effect. This will increase the amount of resources available to each UAV, thereby reducing the overall task completion time. At this point, the timestamps on edges no longer represent actual moments or periods but instead indicate the order or priority of multiple UAVs using service nodes. Therefore, the smaller the timestamp on an edge, the earlier the corresponding path’s UAV will use the resources of that service node compared to other UAVs.

To facilitate understanding, we will construct a directed temporal graph as an example using the research scenario. First, define all available service nodes in the reconnaissance area as the set of vertices $V_{S}=\left\{1,2,\ldots ,10\right\}$ in a directed graph. Next, determine the edges between service nodes based on the order in which UAVs execute tasks. Taking UAV 2 as an example, the service nodes near location point 5 are 5 and 6, and the service nodes near location point 6 are 1 and 7. Therefore, establish directed edges from service node 5 to service node 1, from service node 5 to service node 7, from service node 6 to service node 1, and from service node 6 to service node 7. Label all these edges with the UAV number 2. At the same time, set the weights of these edges to the physical distance between the two service nodes.

**Figure 2:**
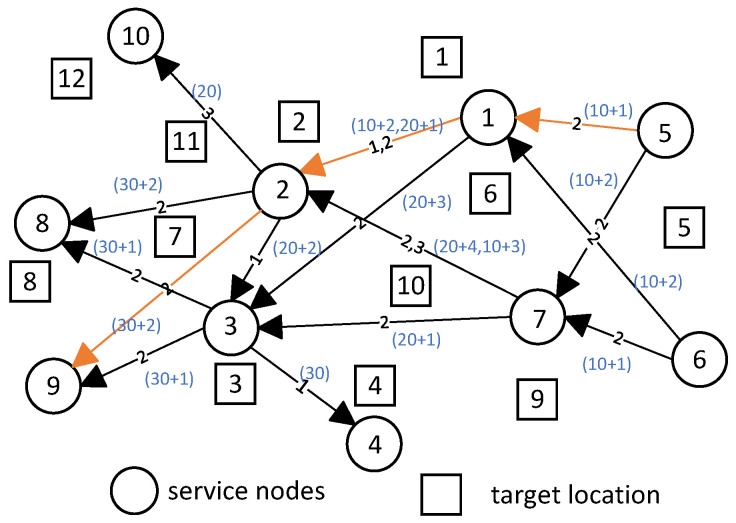
Directed graph corresponding to the research scenario.

The design of timestamps ensures the order in which UAVs execute tasks. Taking service node 2 in Figure 2 as an example, its incoming edge set is $\left\{e_{1,2},e_{7,2}\right\}$. These edges are labeled with 1, 2, and 3, indicating that UAVs 1, 2, and 3 may compete for the use of this service node. When there might be multiple UAVs competing for the same service node, we hope that the service node prioritizes nearby computational demands. For UAV 2, the two potential service needs $e_{1,2}$ and $e_{7,2}$ are at different physical distances. Therefore, we hope to provide computational services first to the demand with a shorter physical distance between the two service nodes, reducing the waiting time of other UAVs through a “first-come, first-served” approach. For UAVs 1 and 2, their service needs are both represented by $e_{1,2}$. At this point, their physical distances are the same, but they involve different types of tasks. UAV 1 has tasks $x_{3}$, $x_{1}$, and $x_{3}$ from service node 1 to service node 2, while UAV 2 has tasks $x_{3}$, $x_{2}$, and $x_{3}$ from service node 1 to service node 2. In the previous discussion, we assumed that UAVs do not need to unload tasks at each location point separately, but can offload multiple tasks at once. Therefore, we assign different weights to different types of tasks here and calculate their total weight. Ultimately, the service node will determine its priority criteria based on two dimensions—the physical distance between service nodes and the total task weight—and provide services for nearby computational demands accordingly.

In the temporal graph construction, we compute the timestamp offsets by considering the “proximity index”, which balances the physical distance between service nodes and task weights. For example, for UAV 2, the timestamp for edge e1,2 is calculated by adding an offset based on the proximity index of tasks ×3 and ×1, which are prioritized based on their respective distances and computational requirements. For edge $e_{i,j}$ labeled with *m*, the method to calculate the sum of task weights $\Lambda_{i,j}$ is within a radius range centered at service node $S_{i}$ or $S_{j}$, select tasks that UAV *m* can execute and sum their weights, resulting in $\Lambda_{i}$ or $\Lambda_{j}$. Thus, the sum of task weights $\Lambda_{i,j}$ for edge $e_{i,j}$ labeled with *m* is defined as $\Lambda_{i,j}=\Lambda_{i}+\Lambda_{j}$. In Figure 2, each edge has its own timestamp. If an edge has multiple labels, it will have multiple timestamps, where each timestamp consists of the sum of a basic quantity and an offset. For example, considering the temporal path 5→1→2→9 with label 2:

1. The basic quantity of timestamp for edge $e_{5,1}$ is 10, and the offset is 1. Thus, the timestamp for edge $e_{5,1}$ is represented as (10+1).
2. The basic quantity of timestamp for edge $e_{1,2}$ is the sum of the previous edge’s basic quantity, 10, and an increment of 10, resulting in 20. The offset is 1. Therefore, the timestamp for edge $e_{1,2}$ is represented as (20+1).

#### 4.1.2. Establish Exclusive Service Network

When solving the task offloading problem, our objective is to reduce the situation where multiple UAVs compete for the same service node, thereby minimizing unnecessary waiting times and the risk of insufficient computational resource allocation. Particularly when there are multiple tasks at each location point, executed sequentially by different UAVs according to a predefined order, it is highly likely that several UAVs will access the same service node near those points. Therefore, we seek a task offloading strategy that can spatially disperse multiple UAVs’ selections of service nodes. Additionally, by assigning different times to different edges, we enable multiple UAVs to access the same service node at different times, thus spreading their usage over time and ultimately achieving dispersed work for UAVs both in space and time. To ensure each UAV’s task offloading strategy is spatially distributed, we will approach this problem from a graph theory perspective. Specifically, this involves solving for non-intersecting paths [28] or finding temporally non-intersecting paths [29]. However, traditional Menger’s theorem does not apply to temporal graphs with timestamps added. Moreover, according to Theorem 4 in [30], the problem of finding multiple temporally non-intersecting paths is NP-complete, meaning no polynomial-time algorithm can be found for an effective solution.

To implement a task offloading strategy that staggers both temporally and spatially, we have designed an offset for timestamps within the temporal graph. When multiple UAVs concurrently compete for the same service node, we assign different offsets based on the proximity index for each UAV. For instance, if the number of UAVs competing for access at service node $S_{i}$ exceeds the number of UAVs competing for access at service node $S_{j}$, then the timestamp offsets for the UAVs competing for $S_{i}$ will be greater than those of the UAVs competing for $S_{j}$. Concurrently, when the base quantities of timestamps are identical, we minimize the timestamp offsets to ensure the spatial dispersion of UAVs, which implies a preference for service nodes with fewer conflicts [31]. We define the traversal time for an edge as the timestamp assigned to that edge, and the total traversal time for a temporal graph path is the sum of the traversal times of all its edges. Therefore, the temporal graph path with the smallest total traversal time is considered the fastest [32].

#### 4.1.3. Search Task Offloading Strategies

After establishing the UAV service network, the next step is to determine the target offloading service node for each task at all location points. We design a two-stage matching algorithm for these independent offloading tasks, achieving overall matching of service node locations, statuses, task types, and offloading demands.

### 4.2. Algorithm Description

In Stage 1, task demands match with temporal paths in the temporal graph. Tasks aim for the most satisfactory offloading, so they select temporal paths based on their preference lists (task type, required resources, priority, and dependencies). Temporal paths, in turn, prioritize tasks with higher demand levels. To ensure task satisfaction and resource efficiency, tasks match with temporal paths of similar demand levels, adjusting the matching between tasks and the temporal graph. In Stage 2, influenced by Stage 1, temporal paths match with service nodes. After determining the task-temporal path matching relationship, temporal paths match with service nodes whose resources meet their demands. Suitable service nodes are those on the shortest path between a task’s start and end points. Our algorithm is shown as Algorithm 1.
**Algorithm 1** Two-stage Matching Algorithm (2-stage MA) based Temporal Graph**lnput**: Predefined action routes *Q* for all UAVs; Distribution of service nodes in the reconnaissance area; Number of UAVs *M*.**Output**: Task Offloading strategy O  1:Initialization: Initialize temporal graph with service nodes as vertices $V_{S}=S=\left\{1,2,\ldots ,S\right\}$  2:**for** each UAV *m* in action routes *Q* **do**  3:      **for** each edges $\left(2^{m},3^{m}\right)\in Q_{m}$ **do**  4:            Create edges between service nodes $S_{k-1}\in S_{2^{m}}$ and node $S_{k}\in S_{3^{m}}$;  5:            Calculate distance $w_{k-1,k}$ between service nodes;  6:            Assign label to edge: ${\mathrm{label}}_{k-1,k}\leftarrow m$;  7:            Set basic timestamp of edge $w_{k-1,k}$ based on path hops;  8:      **end for**  9:**end for**10:**for** each service node with multiple incoming edges $S_{j}$ **do**11:      **for** each edge $\left(S_{i},S_{j}\right)\in E_{S}$ **do**12:            Calculate proximity index for each edge $\left(S_{i},S_{j}\right)$13:      **end for**14:      Sort edges based on proximity index in ascending order;15:      Assign timestamp offsets based on sorted proximity;16:**end for**17:**for** each UAV *m* **do**18:      Determine the set of service nodes near UAV *m*’s first and last location points $S_{1},S_{2}$;19:      **for** each service node $S_{i}\in S_{1}$ **do**20:            **for** each service node $S_{j}\in S_{2}$ **do**21:                  Calculate total traversal time for the path;22:            **end for**23:      **end for**24:      Select the temporal path with minimum traversal time for UAV *m*;25:**end for**26:**for** each tasks $D_{m,k^{m}}$ to be performed by the UAV *m* **do**27:      Send matching requests to all temporal paths that meet task requirements;28:      **for** each $S_{i}\in q_{m}$ (UAV’s selected path) **do**29:            Calculate proximity indices for each temporal path;30:            Select the path with the best utility value;31:      **end for**32:**end for**33:**if** If the available service node’s total resources ≥ task resources required by temporal path **then**34:      Match the task with the service node;35:**else if** the service node’s capacity is exceeded **then**36:      Re-execute step 26 (reassign temporal path)37:**end if**38:**return** the task Offloading strategy O

#### 4.2.1. Establish the Temporal Graph

The steps to define timestamps for edges in a temporal graph are as follows:

1. **Identify nodes with multiple incoming edges**: When a service node has multiple incoming edges, it may need to provide computational services for multiple demands, indicating potential competition for access.
2. **Traverse these nodes and identify any existing conflicts. Sort these conflicts by proximity index to determine the timestamp offsets**: For example, at service node 2, edge $e_{1,2}$ has two labels (1 and 2), and edge $e_{7,2}$ also has two labels (2 and 3). There are four conflict situations in total. Calculate the proximity index for each of these conflicts and sort them from smallest to largest as follows: label 2 of $e_{1,2}$, label 1 of $e_{1,2}$, label 3 of $e_{7,2}$, and label 2 of $e_{7,2}$. Therefore, their timestamp offsets are respectively 1, 2, 3, and 4. The smaller the proximity index, the higher the priority of resolving that conflict, and hence the smaller the timestamp offset.
3. **Identify the service nodes near each UAV’s first location point and set the corresponding outgoing edge basic quantity to 1**: For instance, for UAV 2, the service nodes near its first location point are 5 and 6. Thus, the basic quantity of timestamps for these edges (labeled 2) should be set to 1. Specifically, this includes label 2 of $e_{5,1}$, label 2 of $e_{5,7}$, label 2 of $e_{6,1}$, and label 2 of $e_{6,7}$.
4. **Traverse the directed paths in the temporal graph based on edge labels and determine the basic quantity of timestamps for each edge**: For example, considering the directed path 5→1→3→8, the basic quantities of timestamps on these edges are respectively 10, 20, and 30. Each edge’s basic quantity increases by 10 from the previous one, ensuring that the timestamps in the temporal graph path do not decrease.

#### 4.2.2. Establish Exclusive Service Network

We propose a comprehensive solution: we consider the timestamps (including base values and offsets) of edges as the traversal time for the edges and seek the temporal path with the shortest total traversal time on the temporal graph. Once a temporal path is determined for a UAV, the timestamps on that path are removed. To achieve this by updating the temporal graph such that different UAVs access the same service node at different times. By finding the fastest temporal path for each UAV, we identify the set of service nodes available for selection, thereby constructing their own “computing service network” from the computational resources provided by the network. The procedure to construct this network involves the following steps:

1. Traverse each UAV’s temporal graph path based on the labels in the temporal graph.
2. For a particular temporal graph path, identify $S_{1}$ as the set of service nodes near its first location point and $S_{2}$ as the set of service nodes near its last location point.
3. Find the shortest path from any service node $S_{i}\in S_{1}$ to any service node $S_{j}\in S_{2}$, and calculate the total traversal time for this path.
4. Select the temporal graph path with the smallest total traversal time among all paths as the execution path for the UAV.

### 4.3. Complexity Analysis

This section presents a step-by-step complexity analysis of the proposed algorithm according to the pseudocode. We denoted that *S* is the number of available service nodes, *D* is the number of tasks, *Q* is the number of candidate temporal paths considered by the algorithm, *E* is the number of edges in the temporal graph ($E\leq O(S^{2})$), *L* is the average path length in number of nodes (L≤O(S)), *M* is the number of UAVs and O(·) is the asymptotic upper bound. We assumed that proximity computation between two nodes is O(1) (unless otherwise noted), sorting over *E* items uses a comparison sort in O(ElogE) time, *Q* is retained explicitly in the formulas to show dependency (in the worst case, *Q* can be exponential in *S*, but practical implementations often cap *Q* to keep runtime polynomial.)

1. **Temporal Graph Construction (lines 1–9)**: In this step, we need to compute the pairwise proximity between service nodes, form a candidate edge set, sort edges by proximity, and build graph adjacency structures. Pairwise proximity computation is S2=O(S2). Sort *E* edges is $O\left(E\log E\right)=O\left(S^{2}\log S\right)$ (if $E=O(S^{2})$). Insert edge is $O\left(E\right)=O\left(S^{2}\right)$. So, the time complexity is $O\left(E\log E\right)=O\left(S^{2}\log S\right)$ (if $E=O(S^{2})$), the space complexity is $O\left(E\right)=O\left(S^{2}\right)$.
2. **Phase 1: Task → Temporal Path Matching (lines 10–18)**: In this step, for each task, we need to evaluate *Q* candidate temporal paths to select the optimal or feasible one. For *D* tasks, checking *Q* paths, each of average length *L*, the time complexity is O(D·Q·L) (if L=O(1) (constant-time path evaluation), this reduces to O(D·Q)). Furthermore, if the algorithm needs to enumerate all simple paths in the graph, *Q* may grow exponentially (depending on the graph structure), making this step a bottleneck. In practice, an upper bound is often imposed on *Q* (for example, only taking the shortest paths), in which case *Q* can be treated as polynomial or constant to ensure scalability.
3. **Phase 2: Temporal Path → Service Node Matching (lines 17–25)**: In this step, for each path, we need to check available service nodes for feasibility and assign accordingly. For *Q* paths, each checked against *S* nodes, the time complexity is O(Q·S). Path-to-node mapping and resource status tracking require O(S+Q·L), which is the space complexity.
4. **Objective Evaluation and Finalization (lines 26–37)**: In this step, we need to compute each UAV’s completion time, take the maximum, and check optional conflict and output. The time complexity is O(D+M)≈O(D) (if M≤D).

In conclusion, under the common optimization assumption (if path evaluation takes constant time L=O(1), with $E=O(S^{2})$ and L≤O(S)), the total time complexity further simplifies to $O\left(S^{2}\log S^{2}\right)+O\left(D\cdot Q\right)+O\left(Q\cdot S\right)+O\left(D\right)$. In the extreme worst case, if *Q* grows exponentially with the graph size, the second term O(D·Q·L) will dominate, resulting in an overall exponential complexity for the algorithm, thereby making it non-scalable. The total space complexity is $O(S^{2}+Q\cdot L+D)$.

## 5. Simulation and Analysis

The simulation experiments in this paper ran on a device with an Intel Core i7-14650HX processor and 16 GB of memory, and the simulation was carried out using Matlab R2019a.

### 5.1. Parameter Settings

In a 1 km × 1 km square area, multiple target locations are randomly distributed. There are three types of tasks: $x_{1}$, $x_{2}$, and $x_{3}$, with each location point hosting at least one type of task. The specific locations that each UAV will perform and the types of tasks to be executed at these locations have been predetermined. The distribution of service nodes within the area follows a homogeneous Poisson Point Process (HPPP). The number of base stations is [10,50], the number of location points is [10,50], the range of HPPP intensity variation is [10,40], and the task dependency of each subtask’s indegree is selected between [2,6]. Other parameters of this paper are shown in Table 3.

**Figure 3:**
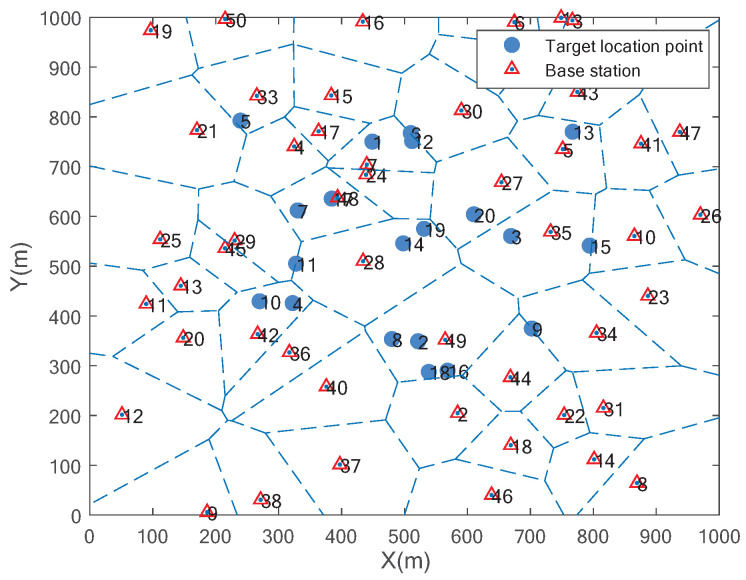
The distribution diagram of each element.

Figure 3 illustrates an example of the simulation scenario, where blue circles represent randomly distributed location points, red triangles represent base stations distributed according to the HPPP, and the numbers near the circles or triangles indicate their respective identifiers. We have compared our proposed algorithm with the Sequential Optimal Matching (SOM) algorithm [33], CBRT [34], and the one-to-many matching algorithm (o2m MA). In the SOM algorithm, each task is sequentially matched to its most preferred service node, which becomes the actual offloading node for the task. In CBRT, the task set is assigned to the service network via the shortest path algorithm, with resource-rich service nodes chosen first. In o2m MA, service nodes, linked to multiple tasks, use the exchange matching algorithm to identify the optimal task offloading strategy. Algorithm performance is assessed based on the total completion time of multi-UAV and task utility. For total completion time, we compare different matching algorithms. For task utility, we compare different task offloading methods.

Moreover, we acknowledge that relaxing the assumption of pre-assigned routes would allow for more flexible UAV operations. Integrating dynamic routing and task allocation is an important direction for future research. Dynamic routing would enable UAVs to adapt their flight paths based on real-time factors such as task urgency and energy levels, while dynamic task allocation would allow tasks to be reassigned during mission execution. Future work will explore integrating these dynamic elements using techniques such as multi-agent reinforcement learning or genetic algorithms.

### 5.2. Performance Analysis

**Figure 4:**
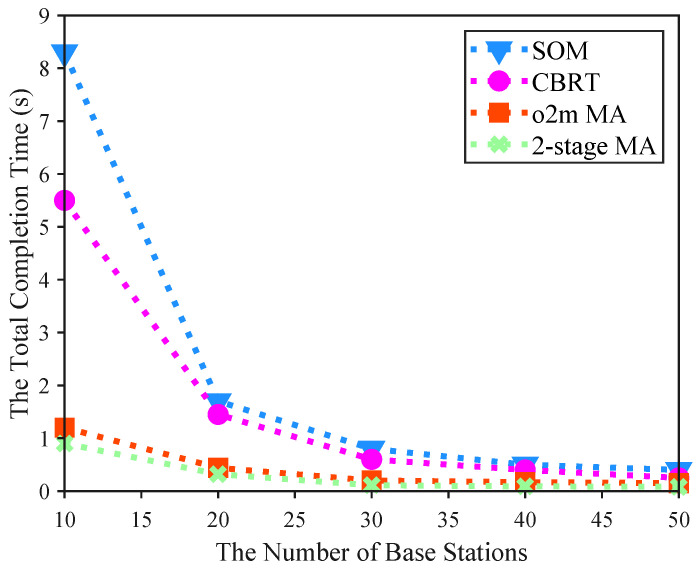
The impact of base station distribution density on the total completion time of the four algorithms.

**Figure 5:**
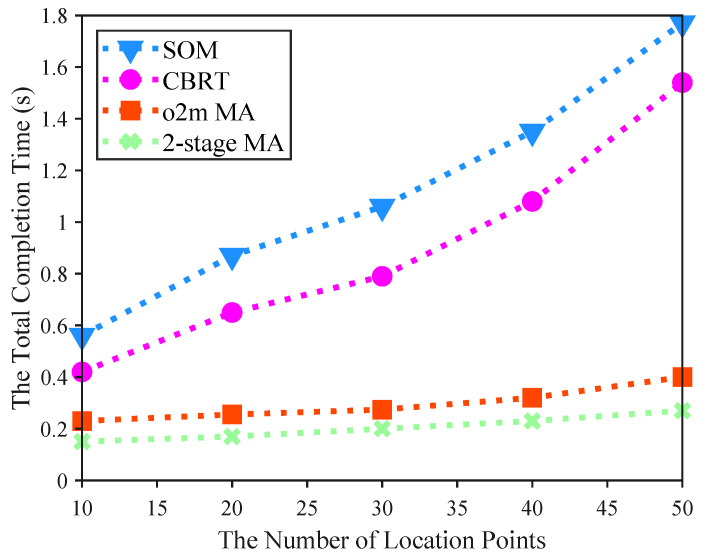
Comparison of the impact of different numbers of location points on the total completion time of four algorithms.

**Figure 6:**
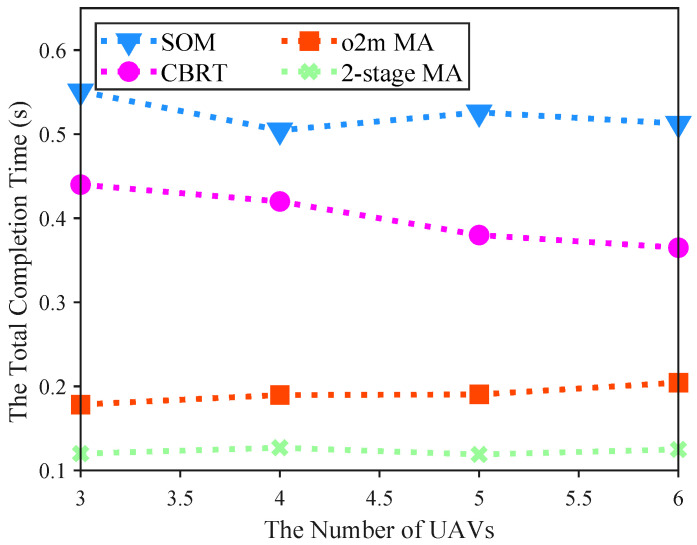
Comparison of the impact of different numbers of UAVs on the total completion time of four algorithms.

**Figure 7:**
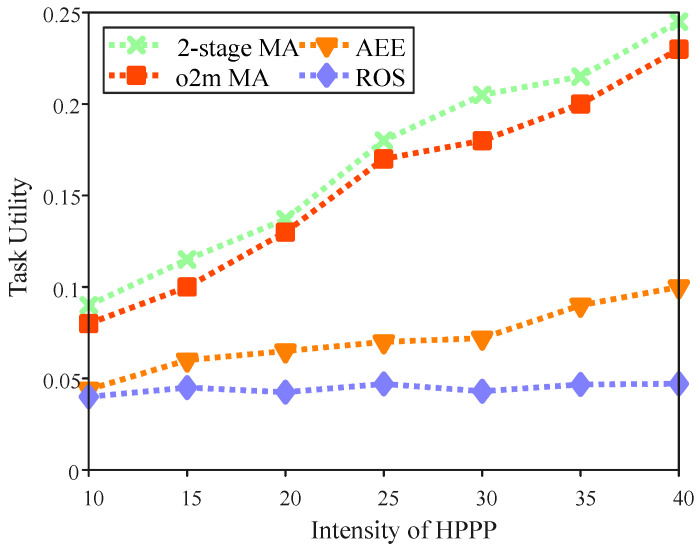
Task utility with the intensity of HPPP.

**Figure 8:**
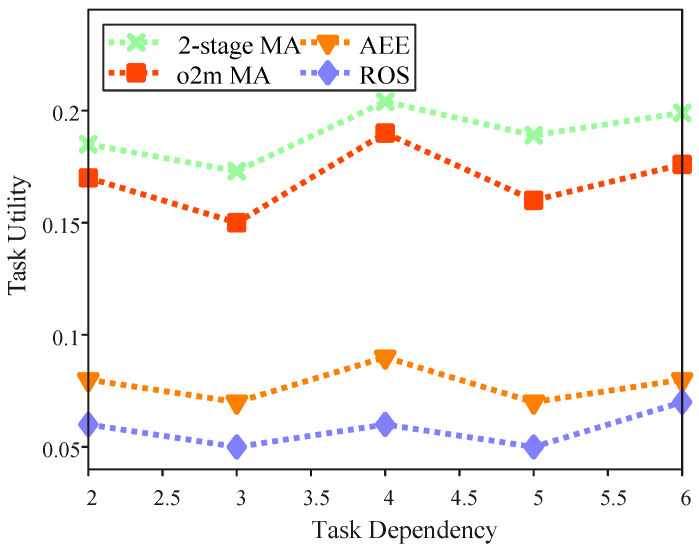
Task utility with the task dependency.

The primary performance indicators of our 2-stage MA algorithm are total task completion time, minimized through optimized temporal graph paths, and task utility, defined as the ratio of successfully offloaded tasks to total task demands. Figure 4, Figure 5 and Figure 6 illustrate the effects of varying the number of base stations, location points, and UAVs on completion time, while Figure 7 and Figure 8 illustrate the impacts of HPPP intensity and task dependencies on task utility. Simulation results demonstrate that the 2-stage MA consistently outperforms baseline algorithms (e.g., SOM [33], CBRT [34], AEE [5], ROS) by achieving significantly lower completion times and higher task utility. This superiority stems from optimized proximity index-based prioritization and two-stage matching, which enhance resource allocation and reduce conflicts via dedicated spatiotemporal networks.

Figure 4 illustrates the impact of varying the number of base stations in the region on the total completion time of UAVs. In this experiment, we configured 20 target location points and 3 UAVs, with the total completion time defined as the maximum among all UAVs, serving as the objective function value. All algorithms exhibit a decrease in completion time as the number of base stations increases, reflecting a negative correlation due to enriched computational resources from more service nodes. Notably, under the same number of base stations, the 2-stage MA consistently achieves shorter times than the others, demonstrating its superior efficiency in leveraging temporal graphs to minimize completion time. For instance, with 30 base stations, the 2-stage MA completes tasks in 0.11 s, compared to 0.7971 s for SOM [33] (approximately 7.25 times faster). Moreover, as base stations increase, the expanded pool of service nodes further reduces delays, amplifying the advantages of the proposed 2-stage MA over baseline methods.

Figure 5 illustrates the impact of varying the number of location points in the region on the total completion time of UAVs. In this experiment, we configured 3 UAVs and 30 base stations within the area. All algorithms exhibit an increase in completion time as the number of location points grows, but the 2-stage MA consistently achieves shorter times than the others under the same conditions. For instance, with 30 location points, the 2-stage MA completes all tasks in 0.2065 s, whereas SOM requires 1.0627 s. This demonstrates the comparative advantage of the proposed 2-stage MA, which maintains significantly lower and more stable completion times, indicating its effectiveness in optimizing task offloading strategies even under increased workload complexity. This superiority is particularly evident in its minimal increase in completion time compared to the substantial rises in the baseline methods (SOM [33], CBRT [34]). The reason lies in the 2-stage MA’s provision of dedicated spatiotemporal networks for each UAV, in contrast to the shared networks used by the baselines like SOM.

Figure 6 illustrates the impact of varying the number of UAVs in the region on the total completion time. In this experiment, we configured 20 target location points and 30 base stations within the area. All algorithms exhibit a general decrease in total completion time as the number of UAVs increases, highlighting improved collaborative capabilities and resource sharing in multi-UAV swarms. Notably, under the same number of UAVs, the 2-stage MA consistently achieves shorter completion times than the other algorithms. For example, with 6 UAVs, the 2-stage MA completes tasks in 0.125 s, compared to 0.5127 s for SOM. The decline is more pronounced in the baseline methods (SOM [33], CBRT [34]), which start from higher times and drop sharply, whereas the 2-stage MA begins at a lower level and remains relatively flat, indicating its high efficiency even with fewer UAVs. The proposed 2-stage MA outperforms the baselines by minimizing task completion times through proximity index-based prioritization and dedicated service networks. This superiority arises because, although all algorithms encounter similar conflicts and competitions with the same number of UAVs, the 2-stage MA employs dispersal strategies in both time and space, thereby reducing conflict probabilities and unnecessary waiting times. Moreover, as the number of UAVs grows, potential conflicts increase, which slightly narrows the performance gap between the proposed algorithm and the baselines.

Additionally, to verify the effectiveness of the proposed algorithm in enhancing task utility, we conducted a comparative study with the following two baseline algorithms and o2m MA (a task offloading strategy based on a matching algorithm that we previously compared):

**All Edge Execution (AEE [5])**: This approach involves assigning all sub-tasks to edge computing resources and selecting the base station with the shortest execution delay as the actual offloading node.

**Random Offloading Scheme (ROS)**: In this scheme, sub-tasks are first sorted in ascending order based on their latency tolerance. Thereafter, a base station is randomly selected as the actual offloading node.

By comparing the proposed algorithm against these baselines, we can assess its performance gains and improvements in task utility.

Figure 7 illustrates the impact of varying HPPP intensity on task utility in the region. In this experiment, we configured 20 target location points, 30 base stations, 3 UAVs, and a task dependency level of 3. All of the algorithms show an increase in task utility as HPPP intensity rises, owing to users’ access to a larger pool of potential offloading nodes, enabling the selection of optimal base stations. This reduces task completion delays and thereby enhances utility. Moreover, at the same HPPP intensity, the 2-stage MA consistently achieves the highest task utility, demonstrating the strongest positive correlation with intensity. This indicates that the proposed 2-stage MA excels in leveraging higher computational intensity for greater utility gains through optimized temporal graph paths, proximity index-based prioritization, and efficient resource matching. It delivers utility improvements of up to 2–5 times over the baselines at high intensities (e.g., approximately 2.84 times better than AEE and 4.77 times better than ROS), by reducing unnecessary waiting, minimizing computation risks, and forming dedicated service networks via temporal graphs. The baselines (AEE [5] and ROS) perform significantly lower; for instance, at an HPPP intensity of 30, AEE achieves 0.0721, ROS achieves 0.043, and the 2-stage MA reaches 0.2049. Overall, this analysis underscores the superior performance of the 2-stage MA in optimizing task utility under varying HPPP intensities compared to the baseline approaches.

Figure 8 illustrates the impact of task dependency on task utility within the region. It explores the relationship between “Task Dependency” (representing the level of sub-task dependencies, where higher values indicate more complex interdependencies between subtasks) and “Task Utility” across four algorithms in a multi-UAV task offloading simulation. In this simulation, we configured 20 target locations, 30 base stations, and 3 UAVs, with an HPPP intensity of 20. Task dependency is defined as the maximum number of direct predecessors for each sub-task. Notably, this value does not follow a clear trend, as the actual number of direct predecessors may be lower than the predefined task dependency level. However, when task dependency remains constant, the 2-stage MA consistently achieves the highest task utility. For instance, when the task dependency value is set to 5, the task utility for AEE is 0.0717, for ROS it is 0.0502, while the 2-stage MA achieves a task utility of 0.189. This demonstrates that the proposed two-stage matching algorithm is more resilient to increasing task dependencies, leveraging temporal graphs to manage priorities, proximity indices, and timestamp offsets for efficient sequencing and resource allocation. These results underscore the superior performance of the 2-stage MA in optimizing task utility at varying levels of task dependency, outperforming other baseline approaches.

Moreover, we justify the restriction to a single task per location based on the complexity of the current model, which was designed to focus on the core aspects of offloading and scheduling. However, we agree that allowing multiple tasks per location could improve system flexibility and better represent real-world scenarios. Future research will explore this by incorporating task prioritization, multi-threaded computation, or task clustering to enable more efficient handling of multiple tasks at each location.

Backhaul latency and interference are important factors that can influence the system’s performance. Backhaul latency, caused by delays in transmitting data between UAVs and MEC servers, can lead to increased task completion times, particularly when UAVs are far from edge nodes or in areas with poor connectivity. Similarly, radio interference between UAVs or from external sources can degrade communication quality, resulting in higher delays and inefficiencies in task offloading.

To assess the potential impact of backhaul latency and interference, we conducted a sensitivity analysis by varying the levels of both factors and observing their effects on task completion time and system efficiency. We set 3 UAVs, 30 base stations, and 30 target location points within the area. As shown in Table 4, task completion time increases and system efficiency decreases as backhaul latency and interference levels rise. Despite these effects, the proposed algorithm maintains reasonable performance under moderate conditions, demonstrating its robustness in the presence of these challenges.

## 6. Conclusions

In this paper, we investigate the task offloading problem in a multi-UAV and multi-MEC environment. When multiple UAVs share computational network resources, we believe that enhancing overall UAV performance can be achieved by either time or spatial dispersion. Initially, we represent the computation network as a temporal graph, with nodes denoting service nodes within the network. Given that different types of tasks at each location point have execution order constraints, we design timestamp offsets for edges to describe task execution sequences through temporal precedence. When service nodes are represented as nodes in the temporal graph, a UAV’s service network appears as a temporal graph path. Based on the number of hops in the temporal graph paths, we assign a base timestamp to each edge. Ultimately, edge timestamps consist of the sum of their offsets and basic quantities. Through this timestamp mechanism, multiple UAVs can use computational network resources at different time intervals, avoiding conflicts. Additionally, to achieve spatial dispersion, we aim for each UAV to select service nodes with minimal conflicts. For this purpose, we adopt total traversal time as an indicator, seeking the fastest temporal graph path for each UAV to determine its exclusive service network. Finally, we employ one-to-many matching theory to address the task offloading problem where both the service node and the task sets are predetermined. Simulation results indicate that the 2-stage MA can provide a dedicated spatio-temporal network for each UAV, demonstrating superior performance compared to the SOM algorithm.

## 7. Future Work

Although our proposed solution performs well in practical scenarios, there remains a gap between the heuristic solution and the theoretical optimal due to the NP-hard nature of the problem. Finding the theoretical optimal solution would require exhaustive computation, which is impractical for real-time UAV swarm operations. Future work could explore metaheuristic algorithms or hybrid approaches that combine exact optimization with heuristics to narrow this gap.

While the current experiments are based on simplified scenarios, we acknowledge that incorporating realistic UAV mobility patterns, energy constraints, and communication uncertainties would enhance the practical applicability of the proposed method. Future work will explore these factors by considering dynamic UAV paths, energy-efficient routing, and communication models that account for interference, signal strength fluctuations, and network congestion. This will help assess the real-world performance and robustness of the algorithm.

## Figures and Tables

**Table 2:** Important notations in this paper.

Symbol	Meaning	Symbol	Meaning
X	Set of tasks	M	Set of UAVs
K	Location set of targets	T	Set of time slots
S	Set of service nodes	Q	Set of routes for all UAVs
D	Set of tasks collected by all UAVs	$x_{n}$	Tasks of type
$\tau_{0}$	Length of time slot	$\mu_{m,k^{m}}$	The computational resources required for the task
$\lambda_{m,k^{m}}$	The input data volume of the task	G	Directed acyclic graph
$V_{D}$	The set of subtasks	$E_{D}$	Dependencies between subtasks
$L_{D}$	Data volume transferred between two subtasks	$\Phi^{\max}$	The maximum cache capacity of the UAV
*R*	The communication rate	*g*	The channel gain
*d*	The distance between UAV and service node	ζ	Path loss
ρ	The connection probability of link	Λ	Task weights

**Table 3:** The simulation parameter settings.

Parameters	Value	Parameters	Value
*M*	[3, 6]	$p_{m}$	100 mW
$\sigma_{0}$	$10^{-10}$ mW	$W_{m,s^{m}}$	[5, 10] MHz
$C_{m,s^{m}}$	[1, 5] GHz	(a,b)	(9.61, 0.61)
$\lambda_{m,k^{m}}$	[500, 800] KB	$\mu_{m,k^{m}}$	[100, 300] MHz

**Table 4:** Sensitivity analysis of backhaul latency and interference on task completion time.

Backhaul Latency (ms)	Interference Level	Task Completion Time (s)	System Efficiency (%)
50	Low	0.131	98.13
50	High	0.168	85.25
100	Low	0.263	95.89
100	High	0.614	82.46
200	Low	0.797	97.20
200	High	1.312	88.34

## Data Availability

The original contributions presented in this study are included in the article. Further inquiries can be directed to the corresponding authors.
